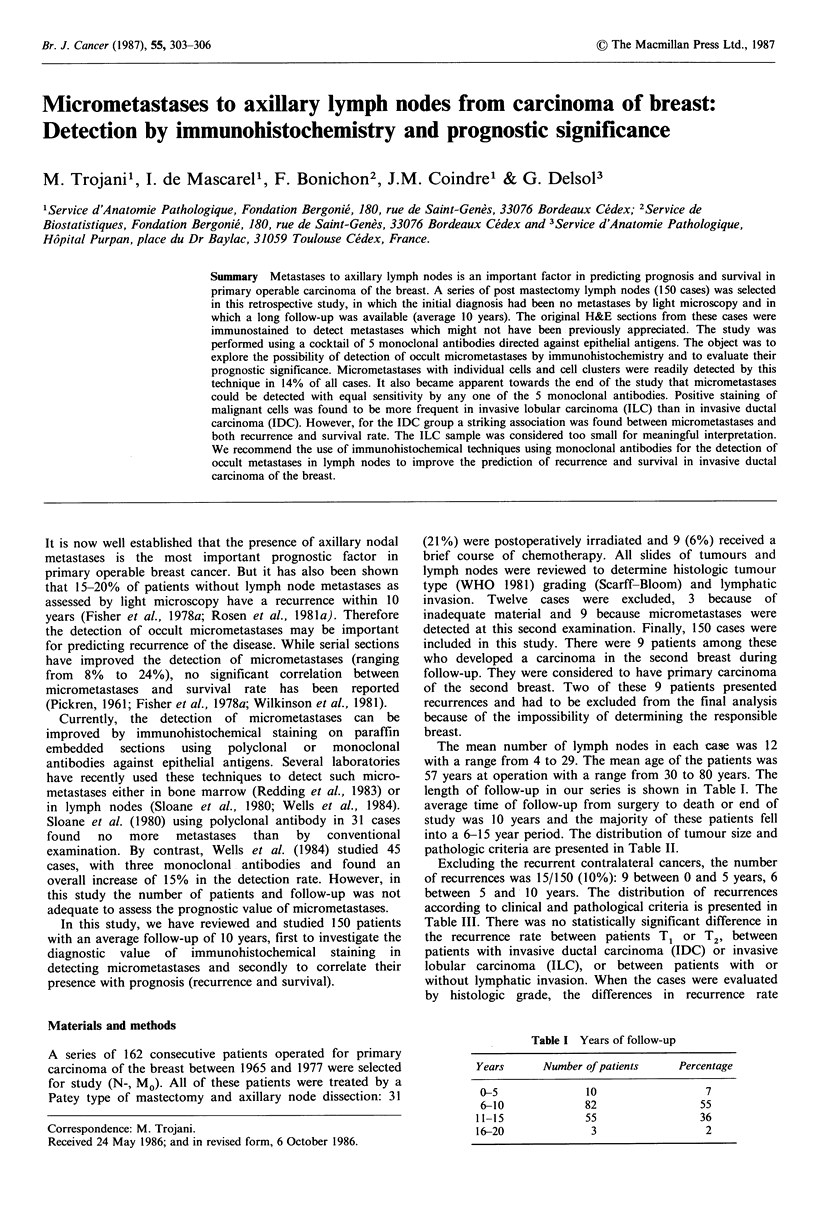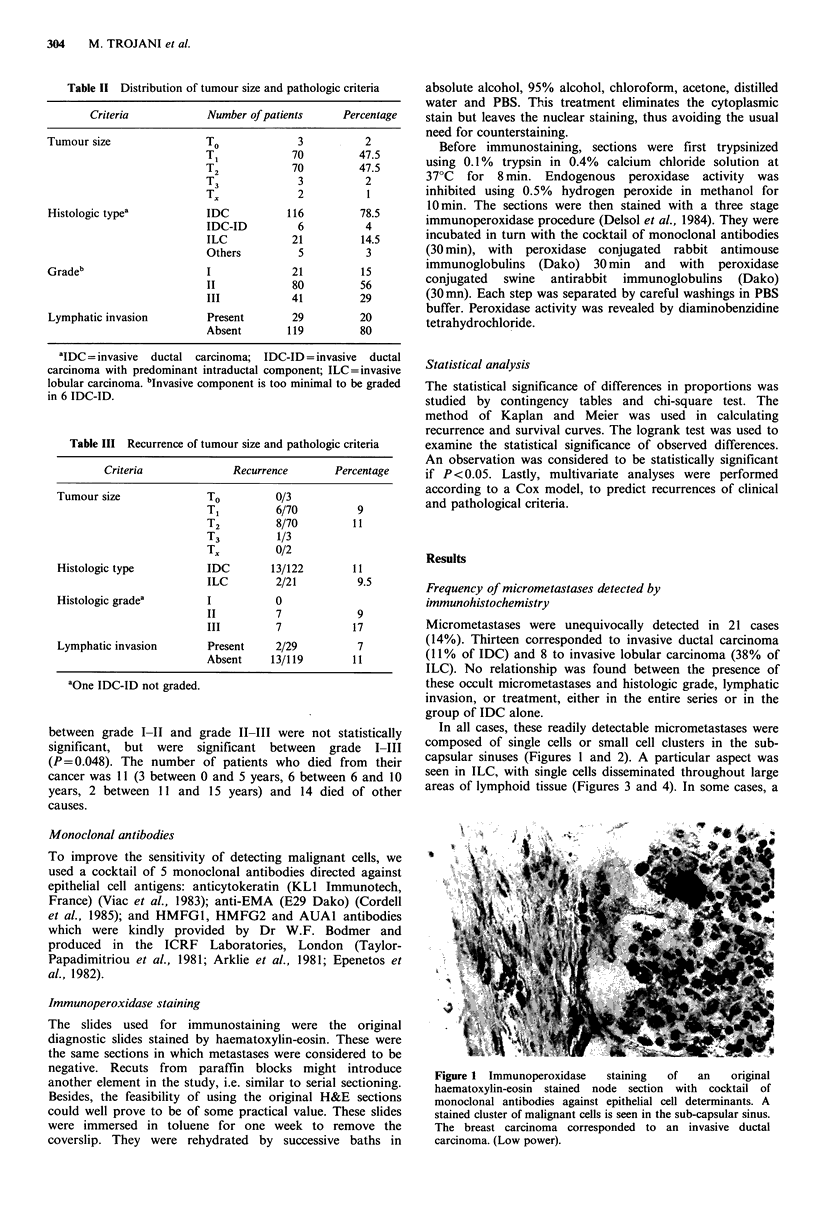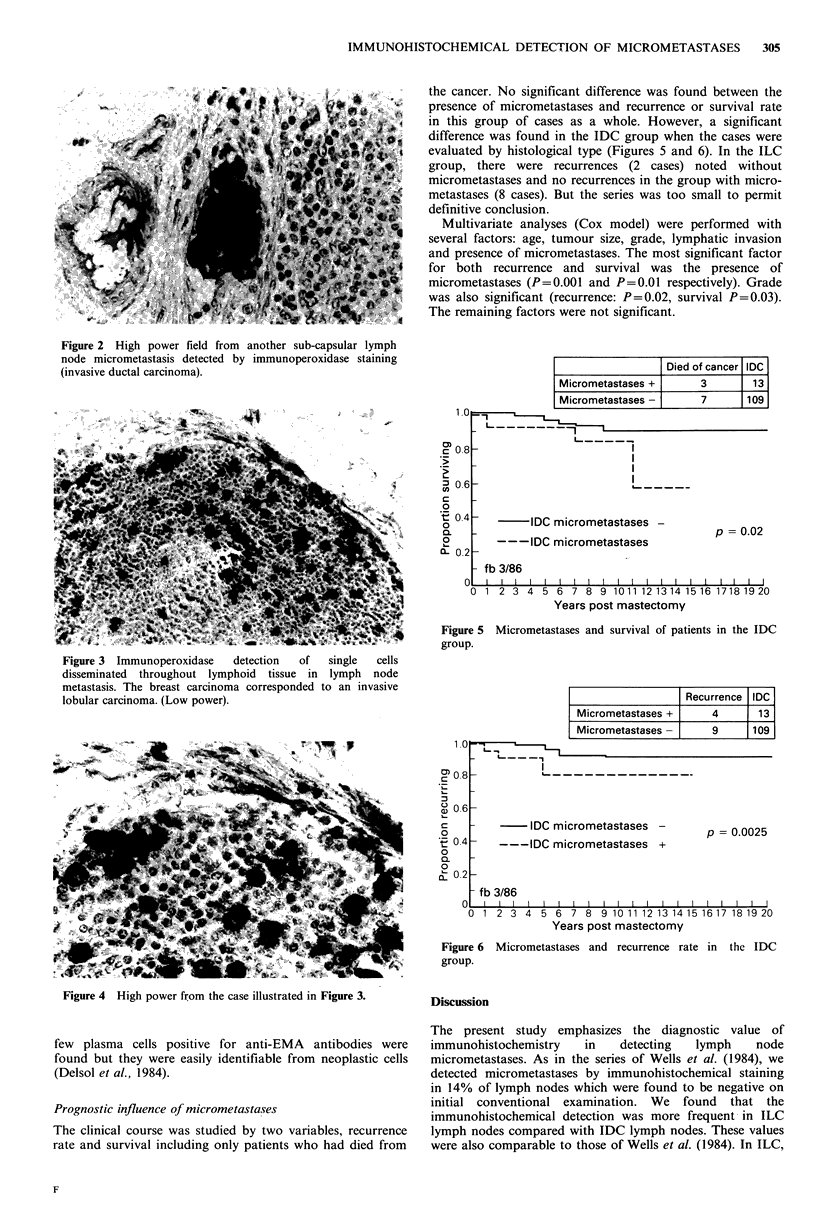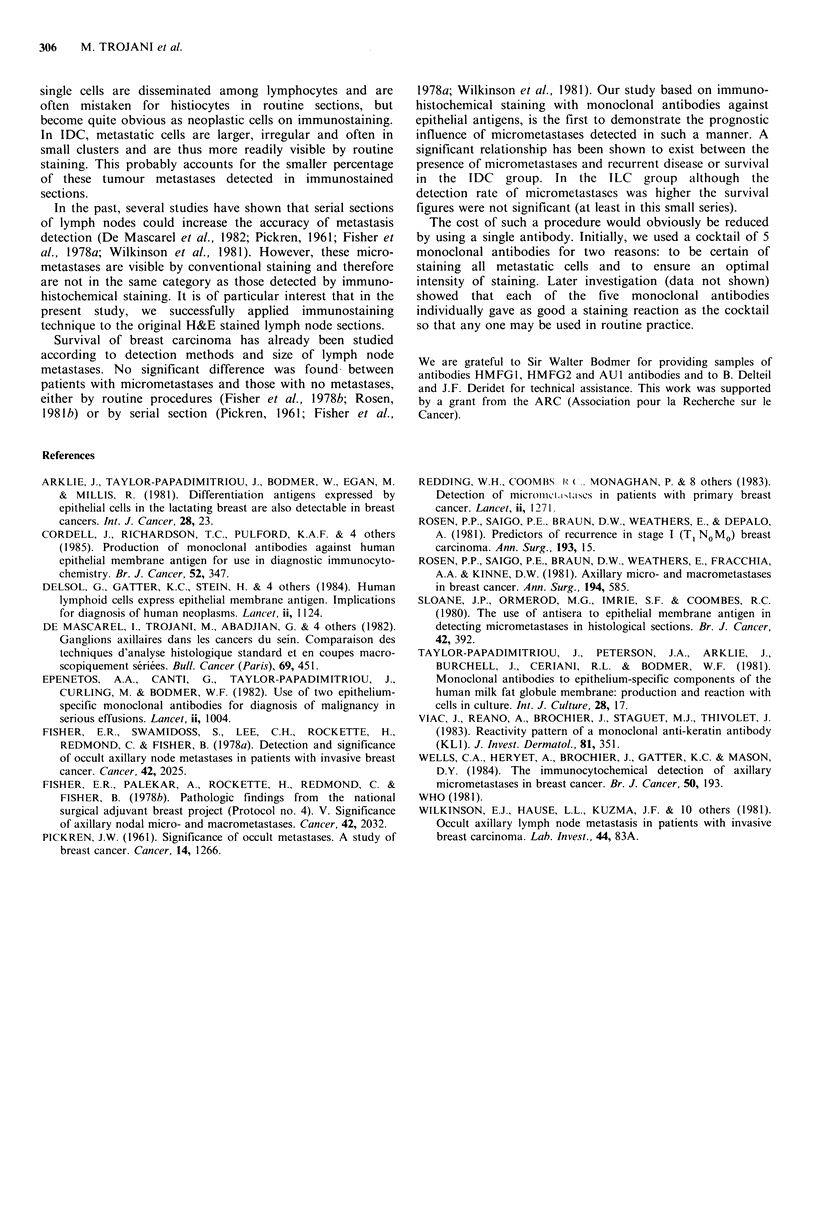# Micrometastases to axillary lymph nodes from carcinoma of breast: detection by immunohistochemistry and prognostic significance.

**DOI:** 10.1038/bjc.1987.59

**Published:** 1987-03

**Authors:** M. Trojani, I. de Mascarel, F. Bonichon, J. M. Coindre, G. Delsol

## Abstract

**Images:**


Br. J. Cancer (1987), 55, 303–306

© The Macmillan Press Ltd., 1987

### Micrometastases to axillary lymph nodes from carcinoma of breast: Detection by immunohistochemistry and prognostic significance

M. Trojani[1], I. de Mascarel[1], F. Bonichon[2], J.M. Coindre[1] & G. Delsol[3]

[1]*Service d'Anatomie Pathologique, Fondation Bergonié, 180, rue de Saint-Genès, 33076 Bordeaux Cédex;* [2]*Service de Biostatistiques, Fondation Bergonié, 180, rue de Saint-Genès, 33076 Bordeaux Cédex and* [3]*Service d'Anatomie Pathologique, Hôpital Purpan, place du Dr Baylac, 31059 Toulouse Cédex, France.*

**Summary** Metastases to axillary lymph nodes is an important factor in predicting prognosis and survival in primary operable carcinoma of the breast. A series of post mastectomy lymph nodes (150 cases) was selected in this retrospective study, in which the initial diagnosis had been no metastases by light microscopy and in which a long follow-up was available (average 10 years). The original H&E sections from these cases were immunostained to detect metastases which might not have been previously appreciated. The study was performed using a cocktail of 5 monoclonal antibodies directed against epithelial antigens. The object was to explore the possibility of detection of occult micrometastases by immunohistochemistry and to evaluate their prognostic significance. Micrometastases with individual cells and cell clusters were readily detected by this technique in 14% of all cases. It also became apparent towards the end of the study that micrometastases could be detected with equal sensitivity by any one of the 5 monoclonal antibodies. Positive staining of malignant cells was found to be more frequent in invasive lobular carcinoma (ILC) than in invasive ductal carcinoma (IDC). However, for the IDC group a striking association was found between micrometastases and both recurrence and survival rate. The ILC sample was considered too small for meaningful interpretation. We recommend the use of immunohistochemical techniques using monoclonal antibodies for the detection of occult metastases in lymph nodes to improve the prediction of recurrence and survival in invasive ductal carcinoma of the breast.

It is now well established that the presence of axillary nodal metastases is the most important prognostic factor in primary operable breast cancer. But it has also been shown that 15–20% of patients without lymph node metastases as assessed by light microscopy have a recurrence within 10 years (Fisher *et al.*, 1978*a*; Rosen *et al.*, 1981*a*). Therefore the detection of occult micrometastases may be important for predicting recurrence of the disease. While serial sections have improved the detection of micrometastases (ranging from 8% to 24%), no significant correlation between micrometastases and survival rate has been reported (Pickren, 1961; Fisher *et al.*, 1978*a*; Wilkinson *et al.*, 1981).

Currently, the detection of micrometastases can be improved by immunohistochemical staining on paraffin embedded sections using polyclonal or monoclonal antibodies against epithelial antigens. Several laboratories have recently used these techniques to detect such micrometastases either in bone marrow (Redding *et al.*, 1983) or in lymph nodes (Sloane *et al.*, 1980; Wells *et al.*, 1984). Sloane *et al.* (1980) using polyclonal antibody in 31 cases found no more metastases than by conventional examination. By contrast, Wells *et al.* (1984) studied 45 cases, with three monoclonal antibodies and found an overall increase of 15% in the detection rate. However, in this study the number of patients and follow-up was not adequate to assess the prognostic value of micrometastases.

In this study, we have reviewed and studied 150 patients with an average follow-up of 10 years, first to investigate the diagnostic value of immunohistochemical staining in detecting micrometastases and secondly to correlate their presence with prognosis (recurrence and survival).

### Materials and methods

A series of 162 consecutive patients operated for primary carcinoma of the breast between 1965 and 1977 were selected for study (N-, $M_0$). All of these patients were treated by a Patey type of mastectomy and axillary node dissection: 31 (21%) were postoperatively irradiated and 9 (6%) received a brief course of chemotherapy. All slides of tumours and lymph nodes were reviewed to determine histologic tumour type (WHO 1981) grading (Scarff–Bloom) and lymphatic invasion. Twelve cases were excluded, 3 because of inadequate material and 9 because micrometastases were detected at this second examination. Finally, 150 cases were included in this study. There were 9 patients among these who developed a carcinoma in the second breast during follow-up. They were considered to have primary carcinoma of the second breast. Two of these 9 patients presented recurrences and had to be excluded from the final analysis because of the impossibility of determining the responsible breast.

The mean number of lymph nodes in each case was 12 with a range from 4 to 29. The mean age of the patients was 57 years at operation with a range from 30 to 80 years. The length of follow-up in our series is shown in Table I. The average time of follow-up from surgery to death or end of study was 10 years and the majority of these patients fell into a 6–15 year period. The distribution of tumour size and pathologic criteria are presented in Table II.

Excluding the recurrent contralateral cancers, the number of recurrences was 15/150 (10%): 9 between 0 and 5 years, 6 between 5 and 10 years. The distribution of recurrences according to clinical and pathological criteria is presented in Table III. There was no statistically significant difference in the recurrence rate between patients $T_1$ or $T_2$, between patients with invasive ductal carcinoma (IDC) or invasive lobular carcinoma (ILC), or between patients with or without lymphatic invasion. When the cases were evaluated by histologic grade, the differences in recurrence rate

**Table I** Years of follow-up

| *Years* | *Number of patients* | *Percentage* |
|---|---|---|
| 0–5 | 10 | 7 |
| 6–10 | 82 | 55 |
| 11–15 | 55 | 36 |
| 16–20 | 3 | 2 |

Correspondence: M. Trojani.
Received 24 May 1986; and in revised form, 6 October 1986.

**Table II** Distribution of tumour size and pathologic criteria

| *Criteria* | *Number of patients* | | *Percentage* |
|---|---|---|---|
| Tumour size | $T_0$ | 3 | 2 |
| | $T_1$ | 70 | 47.5 |
| | $T_2$ | 70 | 47.5 |
| | $T_3$ | 3 | 2 |
| | $T_x$ | 2 | 1 |
| Histologic type[a] | IDC | 116 | 78.5 |
| | IDC-ID | 6 | 4 |
| | ILC | 21 | 14.5 |
| | Others | 5 | 3 |
| Grade[b] | I | 21 | 15 |
| | II | 80 | 56 |
| | III | 41 | 29 |
| Lymphatic invasion | Present | 29 | 20 |
| | Absent | 119 | 80 |

[a]IDC = invasive ductal carcinoma; IDC-ID = invasive ductal carcinoma with predominant intraductal component; ILC = invasive lobular carcinoma. [b]Invasive component is too minimal to be graded in 6 IDC-ID.

**Table III** Recurrence of tumour size and pathologic criteria

| *Criteria* | *Recurrence* | | *Percentage* |
|---|---|---|---|
| Tumour size | $T_0$ | 0/3 | |
| | $T_1$ | 6/70 | 9 |
| | $T_2$ | 8/70 | 11 |
| | $T_3$ | 1/3 | |
| | $T_x$ | 0/2 | |
| Histologic type | IDC | 13/122 | 11 |
| | ILC | 2/21 | 9.5 |
| Histologic grade[a] | I | 0 | |
| | II | 7 | 9 |
| | III | 7 | 17 |
| Lymphatic invasion | Present | 2/29 | 7 |
| | Absent | 13/119 | 11 |

[a]One IDC-ID not graded.

between grade I–II and grade II–III were not statistically significant, but were significant between grade I–III ($P = 0.048$). The number of patients who died from their cancer was 11 (3 between 0 and 5 years, 6 between 6 and 10 years, 2 between 11 and 15 years) and 14 died of other causes.

*Monoclonal antibodies*

To improve the sensitivity of detecting malignant cells, we used a cocktail of 5 monoclonal antibodies directed against epithelial cell antigens: anticytokeratin (KL1 Immunotech, France) (Viac *et al.*, 1983); anti-EMA (E29 Dako) (Cordell *et al.*, 1985); and HMFG1, HMFG2 and AUA1 antibodies which were kindly provided by Dr W.F. Bodmer and produced in the ICRF Laboratories, London (Taylor-Papadimitriou *et al.*, 1981; Arklie *et al.*, 1981; Epenetos *et al.*, 1982).

*Immunoperoxidase staining*

The slides used for immunostaining were the original diagnostic slides stained by haematoxylin-eosin. These were the same sections in which metastases were considered to be negative. Recuts from paraffin blocks might introduce another element in the study, i.e. similar to serial sectioning. Besides, the feasibility of using the original H&E sections could well prove to be of some practical value. These slides were immersed in toluene for one week to remove the coverslip. They were rehydrated by successive baths in absolute alcohol, 95% alcohol, chloroform, acetone, distilled water and PBS. This treatment eliminates the cytoplasmic stain but leaves the nuclear staining, thus avoiding the usual need for counterstaining.

Before immunostaining, sections were first trypsinized using 0.1% trypsin in 0.4% calcium chloride solution at 37°C for 8 min. Endogenous peroxidase activity was inhibited using 0.5% hydrogen peroxide in methanol for 10 min. The sections were then stained with a three stage immunoperoxidase procedure (Delsol *et al.*, 1984). They were incubated in turn with the cocktail of monoclonal antibodies (30 min), with peroxidase conjugated rabbit antimouse immunoglobulins (Dako) 30 min and with peroxidase conjugated swine antirabbit immunoglobulins (Dako) (30 mn). Each step was separated by careful washings in PBS buffer. Peroxidase activity was revealed by diaminobenzidine tetrahydrochloride.

*Statistical analysis*

The statistical significance of differences in proportions was studied by contingency tables and chi-square test. The method of Kaplan and Meier was used in calculating recurrence and survival curves. The logrank test was used to examine the statistical significance of observed differences. An observation was considered to be statistically significant if $P < 0.05$. Lastly, multivariate analyses were performed according to a Cox model, to predict recurrences of clinical and pathological criteria.

### Results

*Frequency of micrometastases detected by immunohistochemistry*

Micrometastases were unequivocally detected in 21 cases (14%). Thirteen corresponded to invasive ductal carcinoma (11% of IDC) and 8 to invasive lobular carcinoma (38% of ILC). No relationship was found between the presence of these occult micrometastases and histologic grade, lymphatic invasion, or treatment, either in the entire series or in the group of IDC alone.

In all cases, these readily detectable micrometastases were composed of single cells or small cell clusters in the sub-capsular sinuses (Figures 1 and 2). A particular aspect was seen in ILC, with single cells disseminated throughout large areas of lymphoid tissue (Figures 3 and 4). In some cases, a

**Figure 1** Immunoperoxidase staining of an original haematoxylin-eosin stained node section with cocktail of monoclonal antibodies against epithelial cell determinants. A stained cluster of malignant cells is seen in the sub-capsular sinus. The breast carcinoma corresponded to an invasive ductal carcinoma. (Low power).

**Figure 2** High power field from another sub-capsular lymph node micrometastasis detected by immunoperoxidase staining (invasive ductal carcinoma).

**Figure 3** Immunoperoxidase detection of single cells disseminated throughout lymphoid tissue in lymph node metastasis. The breast carcinoma corresponded to an invasive lobular carcinoma. (Low power).

**Figure 4** High power from the case illustrated in **Figure 3.**

few plasma cells positive for anti-EMA antibodies were found but they were easily identifiable from neoplastic cells (Delsol *et al.*, 1984).

*Prognostic influence of micrometastases*

The clinical course was studied by two variables, recurrence rate and survival including only patients who had died from the cancer. No significant difference was found between the presence of micrometastases and recurrence or survival rate in this group of cases as a whole. However, a significant difference was found in the IDC group when the cases were evaluated by histological type (Figures 5 and 6). In the ILC group, there were recurrences (2 cases) noted without micrometastases and no recurrences in the group with micrometastases (8 cases). But the series was too small to permit definitive conclusion.

Multivariate analyses (Cox model) were performed with several factors: age, tumour size, grade, lymphatic invasion and presence of micrometastases. The most significant factor for both recurrence and survival was the presence of micrometastases ($P=0.001$ and $P=0.01$ respectively). Grade was also significant (recurrence: $P=0.02$, survival $P=0.03$). The remaining factors were not significant.

| | Died of cancer | IDC |
|---|---|---|
| Micrometastases + | 3 | 13 |
| Micrometastases − | 7 | 109 |

**Figure 5** Micrometastases and survival of patients in the IDC group.

| | Recurrence | IDC |
|---|---|---|
| Micrometastases + | 4 | 13 |
| Micrometastases − | 9 | 109 |

**Figure 6** Micrometastases and recurrence rate in the IDC group.

### Discussion

The present study emphasizes the diagnostic value of immunohistochemistry in detecting lymph node micrometastases. As in the series of Wells *et al.* (1984), we detected micrometastases by immunohistochemical staining in 14% of lymph nodes which were found to be negative on initial conventional examination. We found that the immunohistochemical detection was more frequent in ILC lymph nodes compared with IDC lymph nodes. These values were also comparable to those of Wells *et al.* (1984). In ILC, single cells are disseminated among lymphocytes and are often mistaken for histiocytes in routine sections, but become quite obvious as neoplastic cells on immunostaining. In IDC, metastatic cells are larger, irregular and often in small clusters and are thus more readily visible by routine staining. This probably accounts for the smaller percentage of these tumour metastases detected in immunostained sections.

In the past, several studies have shown that serial sections of lymph nodes could increase the accuracy of metastasis detection (De Mascarel *et al.*, 1982; Pickren, 1961; Fisher *et al.*, 1978*a*; Wilkinson *et al.*, 1981). However, these micrometastases are visible by conventional staining and therefore are not in the same category as those detected by immunohistochemical staining. It is of particular interest that in the present study, we successfully applied immunostaining technique to the original H&E stained lymph node sections.

Survival of breast carcinoma has already been studied according to detection methods and size of lymph node metastases. No significant difference was found between patients with micrometastases and those with no metastases, either by routine procedures (Fisher *et al.*, 1978*b*; Rosen, 1981*b*) or by serial section (Pickren, 1961; Fisher *et al.*, 1978*a*; Wilkinson *et al.*, 1981). Our study based on immunohistochemical staining with monoclonal antibodies against epithelial antigens, is the first to demonstrate the prognostic influence of micrometastases detected in such a manner. A significant relationship has been shown to exist between the presence of micrometastases and recurrent disease or survival in the IDC group. In the ILC group although the detection rate of micrometastases was higher the survival figures were not significant (at least in this small series).

The cost of such a procedure would obviously be reduced by using a single antibody. Initially, we used a cocktail of 5 monoclonal antibodies for two reasons: to be certain of staining all metastatic cells and to ensure an optimal intensity of staining. Later investigation (data not shown) showed that each of the five monoclonal antibodies individually gave as good a staining reaction as the cocktail so that any one may be used in routine practice.

We are grateful to Sir Walter Bodmer for providing samples of antibodies HMFG1, HMFG2 and AU1 antibodies and to B. Delteil and J.F. Deridet for technical assistance. This work was supported by a grant from the ARC (Association pour la Recherche sur le Cancer).

### References

ARKLIE, J., TAYLOR-PAPADIMITRIOU, J., BODMER, W., EGAN, M. & MILLIS, R. (1981). Differentiation antigens expressed by epithelial cells in the lactating breast are also detectable in breast cancers. *Int. J. Cancer*, **28**, 23.

CORDELL, J., RICHARDSON, T.C., PULFORD, K.A.F. & 4 others (1985). Production of monoclonal antibodies against human epithelial membrane antigen for use in diagnostic immunocytochemistry. *Br. J. Cancer*, **52**, 347.

DELSOL, G., GATTER, K.C., STEIN, H. & 4 others (1984). Human lymphoid cells express epithelial membrane antigen. Implications for diagnosis of human neoplasms. *Lancet*, **ii**, 1124.

DE MASCAREL, I., TROJANI, M., ABADJIAN, G. & 4 others (1982). Ganglions axillaires dans les cancers du sein. Comparaison des techniques d'analyse histologique standard et en coupes macroscopiquement sériées. *Bull. Cancer* (*Paris*), **69**, 451.

EPENETOS, A.A., CANTI, G., TAYLOR-PAPADIMITRIOU, J., CURLING, M. & BODMER, W.F. (1982). Use of two epithelium-specific monoclonal antibodies for diagnosis of malignancy in serious effusions. *Lancet*, **ii**, 1004.

FISHER, E.R., SWAMIDOSS, S., LEE, C.H., ROCKETTE, H., REDMOND, C. & FISHER, B. (1978*a*). Detection and significance of occult axillary node metastases in patients with invasive breast cancer. *Cancer*, **42**, 2025.

FISHER, E.R., PALEKAR, A., ROCKETTE, H., REDMOND, C. & FISHER, B. (1978*b*). Pathologic findings from the national surgical adjuvant breast project (Protocol no. 4). V. Significance of axillary nodal micro- and macrometastases. *Cancer*, **42**, 2032.

PICKREN, J.W. (1961). Significance of occult metastases. A study of breast cancer. *Cancer*, **14**, 1266.

REDDING, W.H., COOMBS, R.C., MONAGHAN, P. & 8 others (1983). Detection of micrometastases in patients with primary breast cancer. *Lancet*, **ii**, 1271.

ROSEN, P.P., SAIGO, P.E., BRAUN, D.W., WEATHERS, E., & DEPALO, A. (1981). Predictors of recurrence in stage I ($T_1 N_0 M_0$) breast carcinoma. *Ann. Surg.*, **193**, 15.

ROSEN, P.P., SAIGO, P.E., BRAUN, D.W., WEATHERS, E., FRACCHIA, A.A. & KINNE, D.W. (1981). Axillary micro- and macrometastases in breast cancer. *Ann. Surg.*, **194**, 585.

SLOANE, J.P., ORMEROD, M.G., IMRIE, S.F. & COOMBES, R.C. (1980). The use of antisera to epithelial membrane antigen in detecting micrometastases in histological sections. *Br. J. Cancer*, **42**, 392.

TAYLOR-PAPADIMITRIOU, J., PETERSON, J.A., ARKLIE, J., BURCHELL, J., CERIANI, R.L. & BODMER, W.F. (1981). Monoclonal antibodies to epithelium-specific components of the human milk fat globule membrane: production and reaction with cells in culture. *Int. J. Culture*, **28**, 17.

VIAC, J., REANO, A., BROCHIER, J., STAGUET, M.J., THIVOLET, J. (1983). Reactivity pattern of a monoclonal anti-keratin antibody (KL1). *J. Invest. Dermatol.*, **81**, 351.

WELLS, C.A., HERYET, A., BROCHIER, J., GATTER, K.C. & MASON, D.Y. (1984). The immunocytochemical detection of axillary micrometastases in breast cancer. *Br. J. Cancer*, **50**, 193.

WHO (1981).

WILKINSON, E.J., HAUSE, L.L., KUZMA, J.F. & 10 others (1981). Occult axillary lymph node metastasis in patients with invasive breast carcinoma. *Lab. Invest.*, **44**, 83A.